# OBLIQUE VS. CIRCULAR ANASTOMOSIS IN THE CHILDREN UNDERWENT SOAVE’S PULL-THROUGH SURGERY FOR THE TREATMENT OF HIRSCHSPRUNG’S DISEASE: WHICH IS THE BEST?

**DOI:** 10.1590/0102-672020200003e1545

**Published:** 2021-01-15

**Authors:** Shahnam ASKARPOUR, Mehran PEYVASTEH, Gholamreza DROODCHI, Hazhir JAVAHERIZADEH

**Affiliations:** 1 Pediatric Surgery, Ahvaz Jundishapur University of Medical Sciences, Ahvaz, Khouzestan, Iran; 2 Alimentary Tract Research Center, Ahvaz Jundishapur University of Medical Sciences, Ahvaz, Khouzestan, Iran

**Keywords:** Hirschsprung disease, Constipation, Anastomosis, Surgery, Doença de Hirschsprung Constipação, Anastomose, Cirurgia

## Abstract

**Background:**

Several types of complications including constipation, fecal soiling, perianal excoriation, were reported among different types of surgery for Hirschsprung’s disease.

**Aim:**

To compare circular and oblique anastomoses following Soave’s procedure for the treatment of Hirschsprung’s disease.

**Methods:**

Children who underwent Saove’s pull through procedure with oblique and circular anastomoses were included. Duration of the follow up was two years after surgery. Postoperative complications, such as wound infection, wound dehiscence, peritonitis, fecal soiling, perianal excoriation, were recorded for each patient.

**Results:**

Thirty-eight children underwent oblique anastomoses. Circular ones were done for 32 children. Perianal excoriation was seen in 57.89% and 46.87% of children in oblique and circular group, respectively. Enterocolitis was more frequent in circular (40.62%) than oblique (28.94%) group. Anastomotic stricture was more frequent in circular (15.62%) than oblique (7.89%).

**Conclusion:**

Perianal excoriation was the most common complication among patient in both groups. Oblique anastomoses had fewer complications than circular, and may be appropriate option for patient who underwent Soave’s procedure.

## Graphical Abstract

[Fig f1a]

**Figure f1a:**
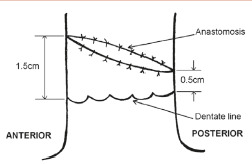
Oblique type of anastomosis

## RESUMO

**Racional:**

Vários tipos de complicações, incluindo constipação, secreção fecal, escoriação perianal foram relatadas entre diferentes tipos de operações para a doença de Hirschsprung.

**Objetivo:**

Comparar as anastomoses circulares e oblíquas realizadas no procedimento de Soave para o tratamento da doença de Hirschsprung.

**Métodos:**

Neste estudo, foram incluídas crianças submetidas ao procedimento pull-through de Saove com anastomoses oblíquas e circulares. A duração do acompanhamento foi de dois anos no pós-operatório. Complicações, como infecção da ferida, deiscência da ferida, peritonite, secreção fecal, escoriação perianal foram registradas para cada paciente.

**Resultados:**

Trinta e oito crianças foram submetidas à anastomoses oblíquas. As circulares foram realizadas em 32. Escoriação perianal foi observada em 57,89% e 46,87% das crianças nos grupos oblíquo e circular, respectivamente. Enterocolite foi mais frequente no grupo circular (40,62%) do que oblíquo (28,94%). A estenose anastomótica foi mais frequente na circular (15,62%) do que na oblíqua (7,89%).

**Conclusão:**

A escoriação perianal foi a complicação mais comum entre os pacientes nos dois grupos. A anastomose oblíqua teve menos complicações do que a anastomose circular e pode ser a opção adequada para o paciente submetido ao procedimento de Soave.

## Graphical Abstract

[Fig f1as]

**Figure f1as:**
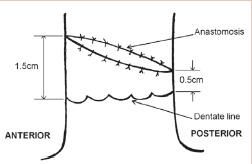
Anastomose oblíqua

## INTRODUCTION

Hirschsprung’s disease which is characterized by the absence of ganglion cell and is a common cause of neonatal intestinal obstruction. Several type of procedures were developed for the treatment of Hirschsprung’s disease such as Duhamel, Soave’s and posterior neurectomy^2^ ^,^ ^7^ ^,^ ^10^. Recent study showed less complication using oblique anastomosis^14^. 

The aim of this study was to compare complications and outcome of patients who underwent circular vs. oblique type of anastomoses for the patients with transabdominal Soave’s procedure.

## METHOD

This retrospective analysis was carried out on the children who underwent transabdominal Soave’s procedure using circular or oblique anastomoses starting from 2013 for five years. Duration of post-surgery follow up was two year. This study was done in Imam Khomeini Hospital of Ahvaz Jundishapur University of Medical Sciences which is the referral center for pediatric and neonatal surgery. This study was approved by research affair of Ahvaz Jundishapur University of Medical Sciences (Registration number=U-98011) and ethical committee of the Ahvaz Jundishapur University of Medical Sciences (IR-AJUMS-1398-059). Patient consent form was signed by parents.

Patients with other perineal or gastrointestinal abnormality, total colonic aganglionosis, with poor follow up, and the ones who underwent laparotomy due to acute abdomen were excluded. Patients with body weight >=10 kg at time of pull-through were included.

**FIGURE 1:**
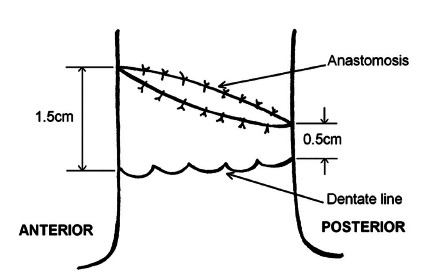
Oblique type of anastomosis

Circular anastomosis was done routinely in Soave’s procedure. In oblique type anastomosis there was 1.5 cm distance between anterior aspects of anastomosis from dentate line while 0.5 cm distance between posterior aspects of anastomosis and dentate line (Figure 1). In the circular type distance between anterior and posterior aspects of anastomosis from dentate line was 0.5 cm.

Patients were categorized into circular and oblique type anastomosis. They were studied in terms of complications such as enterocolitis, constipation, anastomotic stricture, wound infection, fecal incontinency, postoperative fistula, postoperative fever, urologic complication, pelvic infection, wound dehiscence, perianal excoriation, postoperative leukocytosis and mortality.

Duration of postoperative follow up was two years. 

## RESULTS

In the current study 38 children underwent oblique anastomosis and 32 circular anastomoses. Duration of follow up was two years after surgery. Complications of two anastomoses are shown in Table 1. As seen there, enterocolitis was more frequent in circular than oblique type (p=0.004).

Perianal excoriation was the most common complication in both groups, although it was more frequent among cases in oblique group than in circular, but this difference was not statistically significant.

There was no significant difference between two groups in terms of wound infection, length of hospital admission, bleeding during operation, and length of surgery (Table 1). There was no mortality in both groups.

**TABLE 1:** Comparison between oblique and circular anastomoses

Type of complication	Oblique type (n=38)	Circular type (n=32)	p
Enterocolitis	11(28.94%)	13(40.62%)	0.004
Anastomotic stricture	3(7.89%)	5(15.62%)	0.001
Wound infection	8(21.05%)	7(21.87%)	0.120
Duration of hospitalization (mean SE)	7±2.1	8±1.7	0.114
Bleeding during operation (cc)	74±9.4	80±10.3	0.740
Lenght of surgery (minute)	120±10.2	130±8.5	0.845
Peritonitis	1(2.63%)	1(3.12%)	0.002
Complete fecal soiling	0	0	1.00
Postoperative fistula	0	1(3.12%)	0.005
Postoperative constipation	5(13.15%)	3(9.37%)	0.001
Postoperative fever	4(10.52%)	5(15.62%)	0.001
Urologic complication	0	0	1
Pelvic infection	1(2.63%)	1(3.12%)	0.004
Wound or fascia dehiscence	0	1(3.12%)	0.001
Perianal excoriation	22(57.89%)	15(46.84%)	0.084
First postoperative defecation (day)	3±1.2	4±1.8	0.061
First postoperative feeding (hours)	5±2.1	6±1.36	0.072
Leukocytosis after three days of surgery	14(36.84%)	10(31.25%)	0.21
Mortality	0	0	1.00
Second pull-through	0	0	1.00
Soiling after three months of surgery	2(5.26%)	3(6.26%)	0.093
Rectal prolapse	2(5.26%)	1(3.12%)	0.009
Anastomotic leakage	0	0	1.00

## DISCUSSION

Early complications of Soave’s procedure include anastomotic leak, peritonitis, pelvic infection, septicemia, and late complications include strictures, enterocolitis, mucosal prolapse, incontinence and perianal excoriation^4^.

Perianal excoriation was seen in 57.89% and 46.87% of patients with oblique and circular anastomoses. In the study by Pratap et al.^15^ perianal excoriation was seen in 34% of children in pull-through for Hirschsprung’s disease. Perianal excoriation was found in 36.8% and 42% of Shakya et al^16^ and Teitelbaum et al^18^ studies. The higher rate of perianal excoriation in our study may be due to different management in stool frequency and perianal excoriation between centers. Shakya et al.^16^ used coconut oil for perianal excoriation.

Enterocolitis was one of the most frequent complications after Soave’s procedure regardless the type of anastomosis, which is similar to our previous study^1^. Jester et al^5^ showed 12% single episode of enterocolitis after pull-through for Hirschsprung. In the study by Langer^6^ on transanal Soave pulltrough cases, enterocolitis was found in 6%. In the study by Nasr et al.^11^ four of 27 children showed enterocolitis following Soave procedure. The rate of Hirschsprung associated enterocolitis after Soave pull-through was 10% in Prahita et al^13^. In the study by Vega Mata and colleagues^19^ incidence of post- surgery enterocolitis was zero among patients underwent Soave procedure. The rate of enterocolitis in our study was higher than other researches.

Peritonitis was seen 3.12% and 2.63% of children in circular and oblique anastomosis respectively. In the study by Matiolli et al^9^ on children who underwent endorectal pull-through in country with low resource setting, peritonitis was seen in 11 (9.09%) cases. 

Constipation was seen in 13.15% and 9.37% of the children in oblique and circular group respectively. Constipation was reported as a common complication following pull-through in different studies. Widyasari^20^ reported constipation in 24% of children who underwent Soave’s procedure. In our previous publication^1^, constipation was seen in 15% of the cases. In another study constipation occurred in 11.7% of the cases^16^. Constipation may be due to prolonged colonic transit time, postoperative stricture or retained in aganglionic segment^16^.

Fecal soiling was seen in 5.26% and 6.26% of children who underwent oblique and circular anastomosis respectively. In the study by Onishi et al^12^ with more duration follow up that reach 18 year, 18.7% of patients showed incontinence and soiling.

Three of 38 cases (7.89%) in oblique group developed anastomotic stricture. Paul et al^14^ on 17 children, reported one (5.88%) of patients with postoperative anastomotic stricture.

Anastomotic leakage is one of the most serious complication following pull-through surgery. Rate of anastomotic leakage was reported between 1.3% and 8% in different studies^3^ ^,^ ^8^ ^,^ ^17^. In our study, anastomotic leakage was not reported among circular or oblique anastomoses. Anastomotic leakage may be due to technical problem and surgeons experience.

In our study, urologic complication following pull-through was not seen in oblique and circular type anastomoses, as well as mortality. In another study mortality was seen in 5%^16^.

As seen above, oblique anastomosis may reduce complication rates following Soave’s. Similar findings were reported by Paul et al^14^. Here, the rate of some complications, such as anastomotic leakage, was lower than in other studies, but anastomotic stricture and enterocolitis were higher.

The main limitations of this paper are it was done in a single center and with limited sample size. Another multicentric study including a bigger number of patients is recommended

## CONCLUSION

Oblique anastomosis can reduce postoperative complications in contrast to circular anastomosis. 
